# Current Insights into Immunology and Novel Therapeutics of Atopic Dermatitis

**DOI:** 10.3390/cells10061392

**Published:** 2021-06-04

**Authors:** Hidaya A. Kader, Muhammad Azeem, Suhib A. Jwayed, Aaesha Al-Shehhi, Attia Tabassum, Mohammed Akli Ayoub, Helal F. Hetta, Yasir Waheed, Rabah Iratni, Ahmed Al-Dhaheri, Khalid Muhammad

**Affiliations:** 1 Department of Biology, College of Science, UAE University, Al Ain 15551, United Arab Emirates; 201790053@uaeu.ac.ae (H.A.K.); 201970176@uaeu.ac.ae (S.A.J.); 201605261@uaeu.ac.ae (A.A.-S.); mayoub@uaeu.ac.ae (M.A.A.); r_iratni@uaeu.ac.ae (R.I.); 2 Department of Pathology, University of Würzburg, 97080 Würzburg, Germany; Azeem_m@ukw.de; 3 Department of Dermatology, Mayo Hospital, Lahore 54000, Pakistan; dr.attia11@gmail.com; 4 Department of Medical Microbiology and Immunology, Faculty of Medicine, Assiut University, Assiut 71515, Egypt; helalhetta@aun.edu.eg; 5 Foundation University Medical College, Foundation University Islamabad, Islamabad 44000, Pakistan; yasir.waheed@fui.edu.pk; 6 Department of Dermatology, Tawam Hospital, Al Ain 15551, United Arab Emirates; ahmdhaheri@gmail.com

**Keywords:** atopic dermatitis, immune system, T cells, B cells, keratinocytes

## Abstract

Atopic dermatitis (AD) is one of the most prevalent inflammatory disease among non-fatal skin diseases, affecting up to one fifth of the population in developed countries. AD is characterized by recurrent pruritic and localized eczema with seasonal fluctuations. AD initializes the phenomenon of atopic march, during which infant AD patients are predisposed to progressive secondary allergies such as allergic rhinitis, asthma, and food allergies. The pathophysiology of AD is complex; onset of the disease is caused by several factors, including strong genetic predisposition, disrupted epidermal barrier, and immune dysregulation. AD was initially characterized by defects in the innate immune system and a vigorous skewed adaptive Th2 response to environmental agents; there are compelling evidences that the disorder involves multiple immune pathways. Symptomatic palliative treatment is the only strategy to manage the disease and restore skin integrity. Researchers are trying to more precisely define the contribution of different AD genotypes and elucidate the role of various immune axes. In this review, we have summarized the current knowledge about the roles of innate and adaptive immune responsive cells in AD. In addition, current and novel treatment strategies for the management of AD are comprehensively described, including some ongoing clinical trials and promising therapeutic agents. This information will provide an asset towards identifying personalized targets for better therapeutic outcomes.

## 1. Introduction

Skin and subcutaneous diseases are the world’s fourth leading cause of non-fatal disease burden, as stated by the Global Burden of Diseases project [1,2]. Among these, atopic dermatitis (AD) is a substantial contributor of psychosocial and economic burden to the patients and their relatives. The incidence and prevalence of AD have increased over the past several decades [3,4]. It affects up to one fifth of the population in developed countries [5]. Infants are particularly highly susceptible, as AD affects almost 15–20% of children in developed countries. However, AD is also becoming increasingly prevalent among adults [6,7,8]. Clinically characterized by pruritus and eczematous lesions, AD kick-starts the phenomenon of atopic march. Atopic march in infants predisposes them to progressive secondary allergic reactions such as allergic rhinitis, asthma, and food allergies [5,9]. Secondary bacterial skin infections by *Staphylococcus aureus* further enhance the inflammatory responses [10]. Furthermore, sleep deprivation in AD patients owing to pruritus increases the risk of developing attention-deficit/hyperactivity disorder, anxiety, and depression [11,12]. Based on the varying and alternating disease symptoms, recently, four clinical phenotypes of AD were identified. These are, namely, early-onset transient, early-onset persistent, late-onset, and infrequent AD [7,13]. AD can also be classified based on age, phenotype, and cytokine profile, such as pediatric and adult AD, extrinsic and intrinsic AD, and acute and chronic AD. It is also noteworthy that several mouse models for AD have been established as powerful tools for better understanding of the complex pathophysiology of human AD and evaluating the effects of new therapeutic drugs. These models exhibit human AD features such as disrupted skin barrier, pruritus, scratching action, epidermal hyperplasia, and increased serum IgE levels [14].

## 2. Pathophysiology of Atopic Dermatitis

AD is a multifactorial complex disease, so a discrete and definite pathomechanism is still missing. A complex interplay among a disrupted epidermal barrier, itch, skin inflammation, and immune dysregulation along with genetic and environmental factors contribute towards the onset, progression, and chronicity of AD [15]. However, it is not yet clear if epidermal dysfunction precedes immune dysregulation or vice versa. Two theories, namely “inside-out” vs. “outside-in”, have been proposed but remain controversial [16]. The outside-in theory hypothesizes that the inherent damage to the skin barrier function due to disturbed keratinocyte differentiation facilitates the entry of allergens and subsequent immune system activation. On the other hand, the inside-out theory hypothesizes that the immunological cascade that happens as a result of Th2 activation in the skin leads to the AD phenotype [17]. Hence, AD is considered as a biphasic T cell-mediated disease where the acute phase is dominated by Th2 signaling, while a subsequent switch from Th2 to Th1 signaling results in a chronic state of the disease [18]. Th22 cells contribute in maintaining the chronic state of AD with more intense infiltration of T cells, “resident” (CD1c^+^) dendritic cells, and myeloid (CD11c^+^) dendritic cells compared to the acute phase of AD [19].

**Figure 1:**
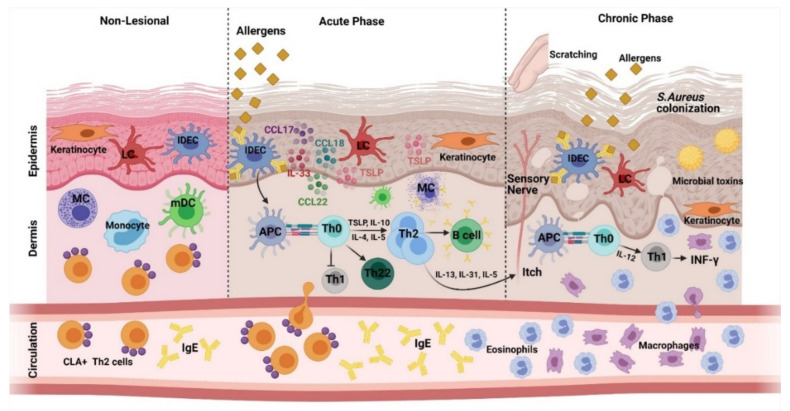
Stage-based pathophysiology of AD indicating various responses and roles of innate and adaptive immune cells during different stages of AD. During the allergic reaction, epidermal dendritic cells with specific IgE bind to the high-affinity receptors of IgE, and Langerhans cells and dermal DC take up allergens and antigens. The type 2 cytokines along with B cells and other Th cytokines directly promote pruritus through sensory nerve activation. Chronicity leads a progressive increase of keratinocyte-derived and various immune-cell-derived cytokines, which further promote itch by various pruritogens.

The outermost layer of the skin is the stratum corneum, which is composed of differentiated keratinocytes called corneocytes. The stratum corneum along with skin surface microbial factors are responsible for maintaining the wholeness of the skin [14]. The pathology of AD (Figure 1) begins when allergens penetrate the disrupted epidermal barrier and hence encounter inflammatory dendritic epidermal cells (IDEC) bearing IgEs; as they have trimeric high affinity IgE receptors, dermal dendritic cells and epidermal Langerhans cells (LCs) are triggered to produce pro-inflammatory cytokines such as thymic stromal lymphopoietin (TSLP), IL-33, CCL17, CCL18, and CCL22 [17]. A sensitization is thus initiated through a T cell-mediated immune response implemented by antigen presentation by professional antigen-presenting dendritic and Langerhans cells [5,14]. Cutaneous inflammation, which is the hallmark of AD, is hence developed [15]. The Th2 response can result in the production of IL-4, IL-13, IL-31, and IL-22, which impairs the skin barrier function further by reducing the expression of epithelial barrier molecules including filaggrin (FLG), LOR, PPL, and claudins [14,17]. Furthermore, IL-4, IL-13, and IL-31 stimulate sensory neurons directly, resulting in pruritus. Scratching due to pruritus induces further activation and infiltration of pro-inflammatory cells, which secrete chemokines such as CCL17 and TSLP, leading towards an enhanced inflammatory response. The disease progresses into a chronic stage with an increase in the role of the Th1 pathway and a persisting contribution of Th2 responses [7,15,20].

## 3. Atopic Dermatitis Phenotypes

Pediatric and adult AD: Even though infants are more vulnerable to AD, its prevalence among adults is also increasing significantly. A recent study revealed a differential T cell polarization in pediatric AD skin and adults [15]. In pediatric AD, the skin is infiltrated by Th17 cells, antimicrobial peptides, Th9/IL-9, IL-33, and other innate markers (IL-1β, IL-8, and IFN-α1) compared to adult AD skin. In adults, AD is the result of Th2 and Th22 polarized cytokine activation in the skin and in blood. T cell activation extends to systemic/cutaneous lymphocytes along with CD8+ and IL-22 producing T cells. However, pediatric AD is Th2 polarized, where such Th2 imbalance is confined to the skin-homing T cell [21]. Furthermore, high blood serum concentrations of IL-31 and IL-33 were found in AD children compared to adults, while no difference in serum concentration of TSLP was observed between the two groups [22].

Extrinsic and intrinsic AD: This classification is based on IgE levels and exposure to environmental factors. Extrinsic or allergic AD is characterized by higher serum IgE along with environmental and food allergen-specific IgE. Intrinsic or nonallergic AD is manifested with normal serum IgE levels and an absence of allergen-specific IgE [23]. Approximately 20% of female AD patients exhibit intrinsic AD. Studies have shown that both intrinsic and extrinsic AD lesions demonstrated upregulated mRNA of Th2 cytokines such as IL-4, IL-5, and IL-13. However, intrinsic AD lesions revealed an enhanced expression of mRNA levels for interferon gamma (IFN-γ) and fork head box protein 3 (Foxp3), the latter of which is a marker for regulatory T cells. Intrinsic AD lesions were also rich in other T cell subsets such as Th17 and Th22 [15,24].

Acute and chronic AD: AD can be differentiated as acute or chronic based on the presiding immune response. The acute phase of AD is dominated by Th2 cytokines such as IL-4, IL-5, IL-22, IL-13, and IL-33, while the immunological mechanism in chronic AD mostly involves Th1/Th0 responses [25]. Studies have revealed that the first 28 h post-allergen exposure results in an immune response predominated by Th2 and Th22 cells, while in long-lasting AD, Th1 cells become authoritative [26]. Th1 cells’ polarization is induced by dendritic cells (DC) [27] when Staphylococcus aureus-derived lipoteichoic acid binds to the toll-like receptor 2 (TLR2) expressed on the surface of the DC, which leads to the production of IL12p70 and IL-23, which in turn amplify Th1 and Th17 polarization [28]. Recent studies have also indicated the contribution of Th17/IL-17 and IL-23 towards the disease’s pathology [14,29,30,31]. IL-23 receptors were upregulated in AD and expressed on several immune cells such as Th17 cells, DCs, and LCs [17,32]. Even though much is not known about the Th17 axis in AD, a study indicated an increase in Th17 cells in acute AD lesions but not in chronic AD lesions [33]. In chronic AD, on the other hand, along with an amplification of the Th2 and Th22 inflammatory responses, there was also more expression of Th1/Th0 cytokines such as INF-γ, IL-6, granulocyte-macrophage colony-stimulating factor (GM-CSF), and IL-12. INF-γ causes keratinocytes’ apoptosis and promotes fierce cutaneous inflammation; IL-12 increases this inflammatory response by upregulating the production of INF-γ and triggering the proliferation of NK cells and T cells [17,34,35].

## 4. Factors Causing Atopic Dermatitis

AD is a multifactorial disease displaying various clinical phenotypes depending on genetic susceptibility, skin barrier dysfunction, environmental factors, and immune dysfunction [16].

Genetic Susceptibility: Recent advancements in molecular medicine have facilitated genome-wide association studies and single nucleotide polymorphism studies [36]. This has led to the identification of 62 different genes that are linked to AD, most of which are genes coding for skin barrier proteins as well as the innate and adaptive immune system [17,37]. The epidermal differentiation complex (EDC) locus on 1q21 consists of a tandem array of gene families that encode proteins responsible for skin cell differentiation. Mutations are reported in genes encoding for skin barrier proteins and genes regulating the differentiation of keratinocytes such as the filaggrin (*FLG*) gene. EDC also contains the loricrin (LOR), claudins, involucrin (IVL), TMEM79/MATT, and SPINK5 genes [17,38]. Mutations recognized in the innate immune system include members of the nucleotide oligomerization domain (NOD)-like receptor (NLR) genes that code for intracellular proteins involved in the regulation of host–pathogen interactions and inflammatory responses such as NOD2, NOD1, TLR2, DEFB1, and CD14. Genetic mutations in genes responsible for Th2 cytokines and chemokines such as IL-4RA, IL-4, TSLP, IL-13, CCR5, and IL-31 are also reported in AD [39]. Variations in genes encoding pattern recognition receptors (PRR) such as TLRs, NLRs, and anti-microbial peptide (AMP) increased the possibility of infection in AD patients [16,40].

Environmental Factors: Climatic variations such as seasonal changes and environmental agents such as allergens and lifestyle contribute towards the onset of AD [16]. Climatic variations that influence AD, especially in children, include low prevalence of the disease during higher mean temperatures and relative humidity with lower precipitation and less indoor heating [41]. Environmental agents also contribute to disease pathogenesis; a study indicated that more than 85% of adults are IgE sensitized to house dust mites (HDMs), thus having specific IgE to HDM although they do not have any clinical allergy. Food allergens were indicated in causing AD flares in children [16,42]. Other environmental factors that enhance AD include high education levels, urban lifestyles with small families, western diets, and excessive use of alkaline soaps [5,16].

Skin Epidermal Barrier Dysfunction: An important function of the skin is to work as a barrier between the internal and external environments of the body. Such epidermal barriers contribute effectively to the internal water retention within cells and prevent leakage of body fluids as well as serving as a biological defense against external stimuli [43,44]. It is thought that various combinations of inherited and exogenous factors accumulate, leading to the breakdown of skin barrier function [5]. The skin consists of a matrix of complex structural proteins and lipids joined by tight junctions and desmosomes. It serves as an efficient microbial, immunological, and physiochemical barrier in which the epidermis exerts several protective functions against external insults and prevents water loss [5,16,45,46]. Barrier dysfunction in AD is a result of deficiency of these components along with a lack of keratinocyte differentiation. Filaggrin (FLG), a keratin-aggregating protein, is one of the important structural proteins in the skin; it is responsible for keratinocyte differentiation, maintaining skin hydration, formation of natural moisturizing factors, cornification, and preventing entry of foreign substances [47,48]. Loss-of-function mutations in the FLG gene are one of the strongest genetic risk factors for developing AD [4,14,49]. FLG polymorphisms are key genetic determinants for AD development along with epigenetic regulation. The polymorphism of other genes related to the immune system and extracellular matrix reinforces the notion of skin homeostasis breakage in AD [36,50,51]. It increases the severity of the disease and susceptibility to infection by *Staphylococcus aureus* and other viral infections [14,52,53]. Furthermore, Th2 cytokines IL-4 and IL-13 also modulate the downregulation of the FLG gene and other epidermal barrier genes in some AD patients [54].

## 5. Innate Immunity in Atopic Dermatitis

AD is characterized by defects in innate immunity and a vigorous adaptive Th2 response to secondary pathogenic infections (see Kuo, et al. [55]). Recent studies have highlighted the importance of the innate immune system, and particularly innate lymphoid cells (ILCs). The Th2-predominant antigen-specific response kicks in after the entry of pathogens as a sequence of skin barrier disruption [56]. However, the antigen-specific T cell response is not much accredited in AD, because the avoidance of only antigen exposure cannot ameliorate AD symptoms. In contrast with the antigen, independent AD has been validated multiple times. A steady state of ILC2, basophils, dendritic cells, and eosinophils in AD skin samples confirms the crucial role of the innate immune system in AD. These components are capable of recognizing pathogen-associated molecular patterns through PPRs and initiating phagocytosis, secretion of antimicrobial peptides, and other pro-inflammatory mediators, resulting in the elimination of the pathogen [57,58,59]. The roles of some of these innate immune cells involved in AD are discussed below.

Dendritic cells (DCs) in AD: Dendritic cells are a well-defined subset of immune cells specialized in antigen uptake and presentation with a vigorous distribution of various DC subsets during different phases of inflammation [60]. There are two important subsets of DCs in the context of AD that show variable expression of different surface markers. One subset is Langerhans cells (LC) CD127^+^ (Langerin^+^), CD1a^+^, and FcεRI^+^, while the other is dermal dendritic cells CD1c^+^, CD11c^+^, and FcεRI^+^. Under inflammatory conditions, another subset develops known as inflammatory dendritic epidermal cells (IDECs) CD1a^+^ CD206^+^. The continuous replenishment of these subsets dependents on the monocytes and recirculating blood pre-DCs [61]. IDECs play a major role in the development of AD, and inhibition of their activation is a focal point of current therapeutics. Jak kinases are important for the development of DCs, and the inhibition of JAK1/JAK2 has been shown to disrupt the development of IDECs from monocytes [62]. The DCs with high-affinity IgE receptors (*FcεRI^+^*) in AD skin are important for initiating the immune response to protein antigens entering the epidermis [21,63]. Plasmacytoid DCs are completely depleted in AD patients due to Th2 cytokine-mediated apoptosis leading to eczema herpeticum susceptibility in AD patients [21,64,65]. Hence, different subsets of DCs play a role during different phases of the disease.

Innate lymphoid cells 2 (ILC2): The increased amount of ILC2 acts as an additional source of Th2 cytokine release in AD lesions [7,66]. Skin residing ILC2 cells are stimulated by IL-25 and IL-33, and in turn they enhance Th2 immune response by releasing IL-13 and IL-5 [6,66]. Keratinocyte-derived IL-25 is essential for the activation of ILC2 and development of skin hyperplasia by IL-13 in multiple skin allergic diseases [67]. However, another study previously indicated that the ILC2 cells are more dependent on TSLP than on IL-25 and IL-33 [68]. Recently, a novel mechanism of interaction between ILC2 and mast cells (MCs) has been reported. MCs induce the production of IL-5 by IL-33-activated ILC2 by miR103a-3p micro-RNA. Co-culture of MCs’ extracellular vesicle (carrying the micro-RNA) with ILC2 showed the upregulation of IL-5 and downregulation of arginine methyltransferase 5 (PRMT5) [69]. Apart from mast cells, basophils are also required for the optimal accumulation of ILC2 cells in AD-like disease. A study also proved that basophil-derived IL-4 promoted the ILC2 response during cutaneous inflammation by enhancing the proliferation of ILC2 cells expressing IL-4Rα in an IL-4-dependent manner [70,71]. Other than ILC2, Sca1^+^Il-10^+^TGF-β^+^ ILC regulatory cells (ILCregs) are also in current focus for therapeutic purposes. It is reported that fucoxanthin can suppress AD symptoms by regulating keratinocytes and ILC regs [72]. However, the roles of ILC2 and ILCregs in AD pathogenesis are still poorly understood and in the very early stages of research.

## 6. Adaptive Immunity in Atopic Dermatitis

AD has been recognized in a systemic form due to the significant presence of activated T cells in the circulation [73]. Epidermal dendritic cells act as a bridge between the innate and adaptive immune systems. DCs play a critical role in the amplification of allergic responses by promoting the Th2 inflammatory axis right from antigen presentation to T cell activation, leading to IgE production by B cells [74,75]. AD is always present with high levels of allergen-specific IgE due to poly-sensitization to various allergens. These IgE antibodies bind to the high affinity IgE receptor FcεRI expressed on the surface of skin DCs, which act as antigen-presenting cells alongside stimulation of other cell surface receptors by microbial antigens and enhance the activation of allergen-specific T cells. This results in a cascade of events leading to the release of soluble mediators in abundance [74,76]. Furthermore, the itch–scratch cycle of AD is amplified through the IgE-mediated histamines released by cutaneous mast cells. The increased skin inflammation is probably mediated by histamine receptors on antigen-presenting cells, keratinocytes, and T cells [77,78]. Such allergic skin inflammation can progress to a response against self-components in the skin, which is also believed to be involved in the pathophysiology of AD [79]. The role of B cells and T cells as effectors of the adaptive immune system in the context of AD are discussed below.

B cells in Atopic Dermatitis: B lymphocytes drive the humoral immune response and maintain it by differentiating into plasma cells that secrete antigen-specific antibodies and memory B cells. They also activate T cells through antigen presentation [80,81]. Activated B cells undergo immunoglobulin class-switch recombination resulting in the production of IgG, IgA, and IgE antibodies [82,83]. This class-switch production of IgE antibodies depends on IL-4/IL-13 signaling regulated by IL-4Rα and STAT6 [84]. During the generation of effective memory B cells, events like class-switching, affinity maturation, and cell differentiation give rise to class-switched memory B cells and long-lived plasma cells [85]. Allergic memory may be maintained by long-lived IgE plasma cells. Furthermore, IgG memory cells serve as the predominant precursors for IgE plasma cells during allergic sensitization and allergic memory responses [84,86]. More recently, a specific subset of B cells is reported to exert a negative regulation on the immune response. Named as regulatory B cells (Bregs), they were shown to have an important role in many autoimmune and inflammatory diseases [87,88]. Even though several subsets of Bregs have been identified based on different markers, their protective effect mainly depends on their capacity to secrete IL-10 [89,90].

Several studies have suggested a role for B cells in the pathogenesis of AD even though it is considered as a T cell-driven disease [91]. The improvement of AD lesions in patients on treatment with a chimeric monoclonal antibody, Rituximab, which depleted the CD20+ B cells, further supports the role of B cells in T cell-driven skin inflammatory diseases [92]. However, whether this role depends on the capacity of B cells to produce antibodies, exert its function on T cells, or both is still poorly understood [91]. High levels of IgE were observed in the blood serum of approximately 80% of AD patients and were associated with increased disease severity through IgE autoreactivity [23]. Another study indicated that IgE plasma cells (CD19^+^ CD38^hi^) and IgE memory cells (CD19^+^ CD38^low^) were increased in the blood of children suffering from atopic diseases [93]. Furthermore, studies have shown that most of the immune cells infiltrating the lesioned AD skin are positive for IgE or its high-affinity receptor *FcεRI* [18,94]. The expression of B cell-intrinsic transcription factor STAT6 is needed during type 2 immune responses for class switch to IgG1 and IgE as well as for GC formation [95,96]. A study on murine B cells indicated that the prolonged activation of the transcription factor STAT6 in B cells during chronic allergic inflammation resulted in IgE responses to viral and bacterial antigens resulting from the subsequent microbial infection that follows the onset of AD. These IgE responses stimulated further activation of mast cells resulting in inflammation [97]. Furthermore, with the help of Th2 cytokines, multiple B cell subsets are activated and differentiated in AD, resulting in significant amounts of circulating IgE [98], some of which becomes autoantibodies [79,99]. Studies have revealed a variety of self-antigens from AD patients, such as manganese superoxide dismutase, keratin 6A, and actin α2, having great homology with environmental antigens [100]. Interestingly, AD severity was correlated with anti-manganese superoxide dismutase IgE autoantibodies [101].

Evidence also suggests that B cells may act as an antigen-presenting cell for T cell activation, especially in response to low antigen concentration [102]. A study on CD19-deficient (CD19^−/−^) mice sensitized by ovalbumin exhibited a histologically weaker AD phenotype along with a lower proliferation of antigen-specific CD4+ T cells and less secretion of Th2 and Th17 cytokines compared to wild type mice. However, adoptive transfer of wild type B cells into these CD19^−/−^ mice resulted in a severe AD phenotype [91,103]. Emerging evidences support the vital protective role of cutaneous B cells in skin homeostasis as well as in the pathophysiology of various skin disorders [104]. Although the exact mechanism of cutaneous B cell migration to skin is not fully defined, various mechanisms involving cutaneous CLA, mediation by different chemokine receptors (namely CCR6, CCR3, CCR7, CXCR3, CXCR4, and CXCR5), and α4β1 integrin- and BAFF-dependent pathways have been proposed in animal studies [105,106,107,108].

The role of regulatory B cells in ameliorating inflammatory allergic conditions is also reported [89]. This data indicates that CD5^+^ CD19^+^ CD1d^hi^ IL-10 producing B cells were much fewer in the splenic CD19^+^ B cell population in an AD-like mouse model, and that IgE secretion was efficiently inhibited by IL-10-producing B cells in normal mice compared to in the AD model. Furthermore, the significant loss of IL-10-producing regulatory B cells in AD led to the defective regulatory function of IgE secretion [109]. IL-10 production from B cells depends on STAT3 activation [110]. This activation is possible through the binding of IL-6 to the CD5 of B cells [111]. In addition, most CD19^+^ CD24^hi^ CD38^hi^ IL-10-producing progenitor B cells are CD5^+^ [112]. Hence, even though IL-6 is thought to play a pathogenic role in AD, it also acts as an inducer of IL-10 secretion by B cells through the CD5–STAT3 pathway, thus exerting a balance and preventing excessive inflammation [113].

The CD24^hi^ CD38^hi^ subset of B cells was characterized as the progenitor of IL-10-producing B cells (B10) in human blood [88,112]. One study that examined the frequencies of CD19^+^ CD24^hi^ CD38^hi^ B10 progenitor cells in AD patients and normal controls indicated that there was no difference in the frequency of B10 progenitor cells between diseased and normal groups [89]. However, the ability of the B10 cells to release IL-10 was significantly reduced in severe AD patients, and to a lesser extent in mild AD patients, compared to normal controls, indicating a negative correlation between IL-10-secreting B cells and AD disease severity [114,115]. The data also supports the fact that IL-10-producing regulatory B cells not only helped in suppressing the allergic disease but also helped in the formation of immune tolerance [113]. Members of the nuclear factor of activated T cells (NFAT) family of transcription factors (TFs), namely NFATc1, NFATc2, NFATc3, and NFATc4, mediate the proliferation and differentiation of peripheral lymphocytes into effector or memory B cells. This is achieved via a Ser/Thr-specific protein phosphatase, calcineurin (CN). As a result of interactions between the immune receptors and the presented antigens, calcineurin is activated, which in turn dephosphorylates NFATs, causing their translocation into the nucleus to activate several immune cell genes downstream [116,117,118]. However, mammalian NFATc1 is expressed in six isoforms in peripheral B-lymphocytes through alternative splicing. While all these isoforms have different functions, NFATc1/αA is a short isoform of NFATc1 predominant expressed during B cell receptor stimulation [119,120]. A few recent studies have shown that NFATc1 transcriptionally inhibits the expression of IL-10 by binding to its gene in association with HDAC1, thus controlling the production of IL-10 by regulatory B cells. Furthermore, ablation of NFATc1 in B cells resulted in more production of IL-10 and reduced imiquimod-induced skin inflammation [121,122].

CLA+T Cells in Atopic Dermatitis: Cutaneous lymphocyte-associated antigen (CLA) is an antigen expressed in 90% of skin-homing T cells and memory T cells [123]; it plays an important role in AD. Studies have shown that during AD, patients tended to have high CLA+ T cells, and the majority of these were memory T cells. The increase of T cell population in AD is not exclusively of CLA+ T cells; a systemic increase of CLA- T cells was also observed. This is an indication of variable effects of AD on patients. CLA+ T cells induce B cells to produce IgE, which prolongs eosinophil survival [2]. In the AD lesions, CLA+T cells are recruited to the skin, which causes inflammation. CLA+ memory T cells tend to increase the production of IL-4, IL-13, and IL-5. These T cells also interact with TSLP and increase the production of IL-4, which prevents CLA+ T cells from going into apoptosis and prolongs the inflammation, causing damage to the keratinocytes [124].

At the molecular level, patients with AD showed decreased DNA methylation in the IL-13 gene compared to healthy patients, which explains the overexpression of IL-13. In addition, reduced methylation was observed in the gene PDE4A, which is a molecule that promotes inflammation. Looking at the micro-RNA (miRNA) expression in CLA+ T cells is of significance in AD, as CLA+T cells of AD patients tend to have an increased expression of miR-21 and miR-145. It was observed that miR-21 was involved in polarization of the adaptive immune responses [125,126,127]. These findings indicate that epigenetic mechanisms also play a role in the pathogenesis of AD by influencing the inflammatory signaling and homing of CLA+ T cells.

Although AD is considered as Th2 predominant, an imbalance of Th2/Th1 immune response is usually observed in patients with AD. This dysregulation of Th2/Th1 explains the immune response in AD, as expressions of Th1 cytokines were detected during AD lesions with an elevation in the expression of IL-4 and INF-γ in mice with AD-like symptoms [128,129]. Th2 cytokines were highly expressed and present in the skin, and an increase in IL-4 was determined in the first 24 h after exposure to the allergen with no expression of INF-γ, which indicated that it is Th2 dominant. However, after 24 h, IL-4 expression decreased and INF-γ expression increased [26]. High frequencies of Th2/Tc2 T cells were also observed in the peripheral blood of severe AD patients [129].

Another Th2 cytokine present in chronic AD patients is IL-31 [130]. Skin biopsies from AD patients have shown a high level of IL-31 expression compared to healthy individuals. A positive correlation was observed among the expression of IL-31, IL-4, and IL-13 mRNA in association with chronic AD [130,131]. One of the Th2 cytokines that downregulate filaggrin is IL-31. It is an inflammatory cytokine often expressed by Th2 CD4+ T cells, and it interacts with the IL-31RA receptor. IL-31 also interacts with immunological and non-immunological cells and tends to have an impact on cell proliferation; it was shown that IL-31 reduces the proliferation of lung epithelial cells. Furthermore, experimental models employing transgenic mice overexpressing IL-31 experienced AD-like symptoms. IL-31 also stimulated the itching sensation, and overexpression of IL-31 in mice increased scratching behavior [132].

In chronic AD, a high population of Th22 infiltrate the skin and are responsible for the production of IL-22, a pro-inflammatory cytokine. It is co-expressed by Th22 and Th17 and was identified to be involved in the pathogenesis of psoriasis [133]. In vitro IL-22 was responsible for slowing down the differentiation of keratinocytes [134]. IL-22 is involved only in severe AD. A study showed that when patients with severe AD were treated with the anti-IL-22 monoclonal antibody *fezakinumab*, the inflammation was reduced [135].

Fibroblasts and Keratinocytes in AD: Defects in keratinocyte differentiation are one of the major factors contributing towards epidermal barrier dysfunction and the onset of AD [47]. Mutations in the *FLG* gene have been implicated wherein filaggrin deficiency results in disorganized keratin filaments, impaired lipid profile, and abnormal bilayer architecture [136]. In steady state, a continuous proliferation, differentiation, and shedding of keratinocytes maintain and repair the skin barrier and function through constant renewal of the epidermis. Such steady state keratinocyte differentiation can be modulated through exogenous triggers, like burning or wounding, and such dysregulation is reported in various skin diseases [137,138]. Fibroblasts interact with keratinocytes via soluble factors such as growth factors (e.g., keratinocyte growth factor/fibroblast growth factor and GM-CSF) as well as interleukins (IL6, IL-13, IL-1) and other secreted factors (e.g., pleiotrophin and stromal cell-derived factor 1). In line with such interactions, various studies have shown putative roles of atopic fibroblasts on keratinocyte differentiation, hyperproliferation, and triggering of inflammatory processes, thereby playing a pivotal role in the pathophysiology of AD [48,139,140,141,142]. Keratinocytes are responsible for pathogen clearance and maintaining tight junction integrity through the production of antimicrobial peptides (AMPs), cathelicidin (LL-37), and human-β-defensins 2 and 3. These are downregulated in AD skin, thus increasing the chances of infection [16,42,143]. On encountering allergens from the environment, keratinocytes secrete thymic stromal lymphopoietin (TSLP), which directly communicates with somatosensory nerves, inducing itch and pruritus [144], and exaggerates Th2-mediated cytokines such as IL-13 and IL-31, which further worsens pruritus by stimulating nerve fibers by upregulating the release of cellular pruritogens [5,145]. TSLP is a pleiotropic cytokine produced by keratinocytes and other epithelial cells that exerts its function on various immune cells such as CD4+ T cells, dendritic cells, ILC2 cells, NKT cells, and mast cells, hence promoting Th2 cell differentiation and Th2 cytokine-associated inflammation [14]. This is achieved by the binding of TSLP to its low-affinity receptor TSLPR to form a high-affinity TSLPR complex comprising a heterodimer of TSLPR and IL-7Rα. TSLP stimulation results in activation of STAT 1, 3, 4, 5, and 6 as well as JAKs 1 and 2 [146]. Studies have shown that TSLP, IL-25, and IL-33 are upregulated by epidermal keratinocytes in lesional AD skin [6]. Keratinocytes also express innate immunity receptors called PRRs, of which Toll like receptor is the most studied [60]. Studies on AD skin have not only indicated that TLR function is reduced in these patients, but also that there is a deficiency of keratinocyte-derived antimicrobial peptides (AMPs) required to limit *S. aureus* and other viral infections, thus predisposing the AD skin to microbial colonization and inflammation [55,147].

## 7. Murine Models for Preclinical Studies of AD

Animal models are an inevitable tool that can be exploited to dissect the complex pathophysiological pathways involved in AD for better understanding of the disease pathology and for development of novel therapeutic agents. Such translational studies using murine models also help to evaluate pre-clinical efficacy and predict dose-to-man [148]. Due to the high phenotypic heterogeneity of AD, a perfect mouse model that embraces all the clinical variants of this disease is not available. However, mouse models showing clinical aspects of AD such as disrupted skin barrier, pruritus, skin hyperplasia, increased serum IgE levels, and scratching behaviors have helped in establishing important insights towards potential therapeutics [14]. Genetically engineered mouse models including transgenic and knockout models are very useful for target validation studies. Transgenic mice are generated by expressing the transgene under the control of a basal keratinocyte keratin (K5/K14) promotor, resulting in a Th2 cytokine profile and dermal infiltration of macrophages, neutrophils, eosinophils, and mast cells [149]. Repeated challenges with haptens such as oxazolone (OXA), dinitrochlorobenzene (DNCB), and trinitrochlorobenzene (TNCB) also induced AD-like phenotypes [150,151]. More recently, AD mouse models induced by topical application of vitamin D3 and its synthetic analogue calcipotriol (MC903) have gained attention [148,152]. It is important to consider that the immunology and skin architecture of mice and human are very different [153], thus limiting the possibility of completely translating the effects of potential drugs from mice to humans.

## 8. Therapeutic Strategies in Atopic Dermatitis

Currently, no complete cure is available for AD, and the only way to manage the disease is by palliative treatment. There are various substances to treat AD—from topical treatments and immunosuppressive agents to biologics, which are detailed below. The supportive therapies are aimed at avoiding secondary infections and improvement of skin integrity [154]. Even though a guideline-based treatment strategy is still not available for controlling AD, efficient clinical management usually involves basic management and step-up treatment [155]. Basic management involves methods such as skin hydration, allergen avoidance, elimination of triggers, and wet wrap therapy (WWT). Step-up treatment inculcates additional treatment methods. In this method, mild to moderate AD is managed by topical anti-inflammatory therapies, such as topical corticosteroids or topical calcineurin inhibitors, for treating acute flares or maintenance treatment [156] along with adjuvants such as antihistamines [157] and treatments for bacterial, fungal, and viral infections. On the other hand, treating moderate to severe AD is more challenging; it requires additional systemic treatment strategies such as biologics, immunomodulating agents, allergen immunotherapy (AIT), anti-IgE therapy, and in worst cases phototherapy [158] and hospitalization. In other words, there are three lines of therapeutic strategies that are currently implemented for the management of atopic dermatitis. This includes first line therapies involving basic management; second line therapies involving basic management with step-up treatment with topical medications and adjuvants; and third line therapies involving basic management, topical medications, and systemic medications, along with phototherapy and allergen immunotherapy in the worst cases.

### 8.1. First Line Therapies

The possible chances of following up with the required therapies are very meagre, since the treatment strategies for AD demand a substantial contribution from patients and their families. Hence, educating the patient about their skin condition is the first and most important part to be fulfilled to provide efficient healthcare support, especially in pediatric patients [159,160]. Furthermore, an effective treatment plan is mostly successful through the collaboration of an inter-professional medical team comprising dermatologists, psychologists, dieticians, and nurses towards addressing various aspects of the disease such as the medical requirements and mental and behavioral factors [160,161]. The first line therapies include basic management such as skin hydration, allergen avoidance and elimination of triggers along with some step-up treatments involving topical agents to overcome acute flares or maintenance treatment.

AD is a chronic, inflammatory, and relapsing disease. Hence, long-term maintenance therapy is preferred and recommended over treatment in response to flare-ups. The skin of AD patients always exhibits subclinical epidermal barrier defects and inflammatory signs. This requires an application of emollients or moisturizers even on unaffected areas daily. A generous application of moisturizers is highly recommended for patients suffering from mild, moderate, or severe AD. Some evidence suggests that the persistent use of moisturizers from birth is a fruitful method to prevent the onset of AD in high-risk infants [160]. This helps in controlling flare-ups and diminishing the associated pruritus and xerosis, hence reducing the need to use topical steroid preparations [162]. Moisturization reduces trans-epidermal water loss (TEWL), thus maintaining the skin moisture required for proper repairing of the damaged skin barrier. These moisturizers maybe delivered in the form of cream, lotion, or ointments depending on patient preference, site of application, climate, and extent of skin dryness [160,163].

It has been well validated that the first line of therapy for inflammatory symptoms such as acute flares and itchiness of AD is achieved by topical corticosteroids (TCSs) in both adults and children [164]. However, the continuous application of topical corticosteroids should be avoided, as it has several adverse effects such as adrenal suppression, skin atrophy, perioral dermatitis, etc. Hence, patients should reduce its use once the symptoms have subsided before starting with maintenance therapy [165]. On the other hand, tacrolimus and pimecrolimus are two nonsteroidal topical calcineurin inhibitors (TCIs) that do not have the long-term harmful effects of TCSs and can also safeguard the skin barrier weakened by prolonged use of topical steroids. However, these TCIs may cause momentary itching or burning sensations at the site of application [160]. While studies have suggested that 0.1% tacrolimus ointment in combination with TCSs is effective and safe for long-term treatment of moderate to severe AD in both children and adults [166,167], a 1% pimecrolimus ointment is considered effective and safe for mild to moderate AD in both adults and children [160,168]. Furthermore, even though studies on mouse models revealed a possibility of developing lymphoma on exposure to high doses of TCIs, such adverse effects were not seen in humans [169]. Hence, these prefatory treatment strategies can help contain acute AD flares and/or maintain a passive state of the disease.

### 8.2. Second Line Therapies

The chronic AD condition can lead to other complications such as bacterial, fungal, and viral superinfections. It is especially well established that AD skin lesions are very susceptible to colonization by *S. aureus*, which can further worsen the itch by inducing pruritus. Hence, a short-term use of topical and/or antimicrobial therapies is considered as the second line of treatment strategy, since continuous use of such medications may lead to antibacterial resistance [160]. One study indicated the use of first generation cephalosporins and the implementation of warm water baths with non-irritating mild acid soaps to be effective against *S. aureus* colonization. While cephalosporin is an efficient antibiotic, the antiseptic properties of bleach also help to control *S. aureus* infections in AD patients [10]. However, some clinicians prefer to also prescribe antibiotics with anti-inflammatory properties such as doxycycline and cortimoxazole. Superinfections caused by viruses such as the herpes simplex virus are often treated with antivirals such as acyclovir or its derivatives. Fungal infections such as from *M furfur* can be treated with antifungals containing azole agents either topically or systemically [160]. Interestingly, a few studies have shown that supplementing pregnant mothers or mothers of infants with probiotics such as Lactobacillus alone or Lactobacillus with Bifidobacterium can delay the onset of AD up to 20% in the first three years of life [170,171,172].

Histamines are released in response to scratching action and worsen pruritus. This disturbs the patient’s sleep quality, so antihistamines are also used in second line therapies. Moreover, the use of oral sedating with, e.g., hydroxyzine, diphenhydramine, or doxepin is also in practice [173]. It is well known that IgE-mediated food allergies are widespread in almost one third of AD patients. The most popular foods implicated in such allergies include peanuts, wheat, egg, milk, and soy [160]. An increase in phosphodiesterase 4 (PDE4) levels in response to raised production of several chemokines and cytokines is elicited in AD flares. Crisaborole is a promising non-steroidal anti-inflammatory agent that is a PDE4 inhibitor. This inhibitor drug helps reduce the levels of pro-inflammatory cytokines and chemokines by upregulating adenosine monophosphate levels intracellularly, thus inhibiting the NF-κB pathway responsible for release of pro-inflammatory cytokines such as TNF-α [174,175]. Phase III clinical trials have proved that a 2% ointment of this compound has good efficacy and safety in both adult and pediatric patients suffering from mild to moderate AD [176].

### 8.3. Third Line Therapies

**Figure 2:**
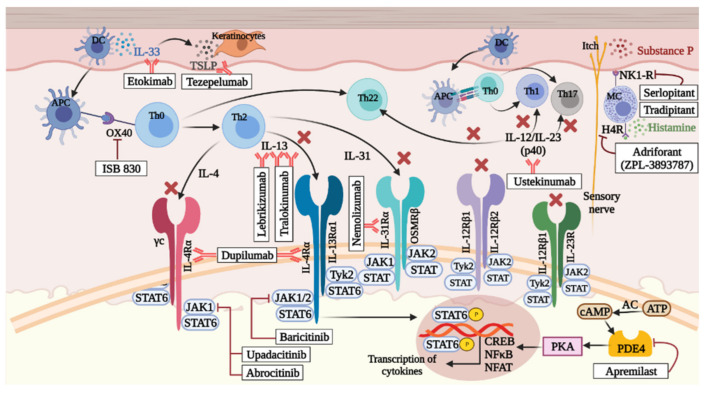
A summary of various therapeutic biologics and small molecules used to treat AD.

Failure of improvement from the first or second line of therapies in patients suffering from chronic and severe AD requires an additional third line of systemic therapeutic strategies. Various third line therapeutic options are detailed below and summarized in Table 1 and Table 2 and Figure 2.

#### 8.3.1. Emerging Therapeutic Biologics for AD

In recent years, various newly developed biologics have been approved as well as put under clinical trials by the Federal Drug Agency (FDA) and the European Medicines Agency (EMA) (see overview in Table 1 and Table 2). Patients with AD benefit from their high efficacy. Details of such molecules are briefly discussed below (see also Figure 2).

Dupilumab, a human monoclonal IgG4 antibody targeting the alpha subunit of IL-4 receptor, is probably the most promising systemic therapy for AD, as inhibiting the IL-4 and IL-13 signaling pathways is an efficient mechanism for controlling the Th2 response [7,160]. It reduced serum levels of CCL17, an AD biomarker and a key regulator of Th2 response. It was approved in 2017 for treating adults with moderate to severe AD and showed significant betterment of pruritus and inflammation without showing any dose-limiting toxicity [177]. Dupilumab was shown to change the AD transcriptome, helping in skin normalization by downregulating genes of inflammatory mediators and markers of epidermal proliferation while upregulating genes of structural proteins, epidermal barrier proteins, and lipid metabolism proteins [178].

Lebrikizumab is an anti-IL-13 monoclonal antibody. IL-13 has been observed to be overexpressed in AD skin lesions, contributing to AD pathogenesis. Excess IL-13 has been shown to downregulate genes coding for crucial structural proteins such as loricrin and involucrin. Lebrikizumab helps to inhibit IL-13 production by preventing the formation of the IL-13Rα1/IL-4Rα heterodimer receptor-signaling complex. However, it does not prevent the binding of IL-13 to its decoy receptor IL-13Rα2 required for the endogenous regulation of IL-13. Lebrikizumab was shown to have efficacious results improving quality of life in adult patients with moderate to severe AD in terms of pruritus and skin lesions in a dose-dependent manner [179,180].

Tralokinumab is a human IgG4 monoclonal antibody capable of binding to IL-13, thus preventing the interaction with its receptors and following signaling cascade. Tralokinumab was shown to reduce *S. aureus* colonization on AD skin in patients with moderate to severe AD, probably by enhancing skin antimicrobial responses and improving skin barrier integrity [181].

Nemolizumab is a human monoclonal antibody targeting IL-31α subunit, which contributes towards AD pathogenesis. IL-31 is responsible for causing pruritus in AD. It is expressed mainly by Th2 lymphocytes and functionally targets a variety of immune cells such as basophils, eosinophils, and monocytes along with epithelial cells and keratinocytes. Clinical trials have shown that nemolizumab has good efficacy in reducing pruritus and skin inflammation [180,182].

Fezakinumab is an anti-IL22 monoclonal antibody. IL-22 is a significant contributor to AD, causing epidermal hyperplasia by promoting keratinocyte proliferation and barrier defects by promoting inhibition of terminal differentiation [135]. Blockade of IL-22 by fezakinumab helped in ameliorating epidermal responses in AD patients. Furthermore, treatment with fezakinumab also resulted in inhibition of a multitude of inflammatory mediators secreted by IL-22 receptor (IL-22R)-expressing keratinocytes [135,183]. Currently, this drug is not undergoing clinical trials for AD [184].

Ustekinumab is a human IgG1 monoclonal antibody that inhibits IL-12 and IL-23 by blocking their common subunit p40 and thus significantly reduces Th1 and Th17/Th22 responses. It is commonly used to treat psoriasis by reducing skin inflammation. However, some phase II clinical trials show great efficacy of ustekinumab in the treatment of moderate to severe AD patients [185,186].

Etokimab is a human IgG1 monoclonal antibody that inhibits IL-33, an important player in AD pathogenesis. IL-33 contributes towards inflammation in AD through the IL-33/ST2 signaling pathway, thus initiating innate and adaptive type 2 responses. A phase II clinical trial with etokimab revealed that it effectively blocked IL-33 that acted upstream of the inflammatory signaling cascade and hence modulated neutrophil migration directly and indirectly through inhibitory effects on the IL-8 pathway [187]

Omalizumab is a recombinant humanized IgG1 monoclonal antibody against IgE. It binds to the Fc (CH3 domain) of IgE molecules and prevents the interaction with its high-affinity FcεRI receptor, as the CH3 domain serves as the binding site to its receptor. High levels of IgE are associated with the severity of AD. Moreover, pediatric AD patients exhibit more IgE involvement. Omalizumab has already been FDA approved to treat asthma in children above 6 years. Clinical trials of omalizumab to treat AD in child patients showed promising results in improving the quality of life and reducing AD severity [180,188,189]. However, this drug is not currently undergoing clinical trials for the treatment of AD [184].

ISB 830, previously known as GBR 830 [184], is a human IgG1 monoclonal antibody specific for OX40. It binds to OX40 (CD135), a co-stimulatory molecule expressed on T cells, and prevents the interaction with its ligand OX40L (CD252), thus inhibiting the potentiation of T cell responses triggered through T cell receptors. A clinical trial on moderate to severe AD patients with ISB 830 was well tolerated, and on examining the AD lesions, a decrease in Th1/Th2 and Th17/Th22 mRNA was observed, indicating an effect of ISB 830 on both acute and chronic AD [190].

Tezepelumab is an IgG2λ human monoclonal antibody targeting thymic stromal lymphopoietin (TSLP). AD patients show increased levels of TSLP in their skin lesions. TSLP helps in promoting inflammatory responses through several pathways involving mast cells and dendritic cells. TSLP acts by binding to its receptor TSLPR and forming a heterodimer with IL-7 receptor α to polarize dendritic cells towards the expansion of Th2 cells, ILC2, basophils, and other immune cells associated with type 2 immune response. Tezepelumab binds to the TSLP receptor and prevents the interaction with its receptor, thereby blocking all downstream inflammatory pathways. Phase II clinical trials with tezepelumab showed limited efficacy and statistically insignificant clinical improvement. Further studies may be required with longer treatment periods for better treatment effects [191,192,193,194].

#### 8.3.2. Emerging Therapeutic Small Molecules for AD

Baricitinib is a first generation JAK1/2 inhibitor. It has already been approved for the treatment of other inflammatory diseases such as rheumatoid arthritis. Clinical trials of baricitinib for treating AD showed promising results in reducing clinical signs of AD and relieving pruritus [195,196].

Abrocitinib is a second generation small molecule inhibitor that selectively inhibits JAK1 associated with receptor chains, which dimerizes on receptor activation to form receptor complexes. Several cytokines involved in the pathogenesis of AD activate JAK1-containing heterodimeric receptors, resulting in downstream itch signaling and Th2 differentiation. Hence, selective inhibition of JAK1 helps in regulating a wide range of inflammatory cytokines without causing adverse effects of JAK2 inhibition such as neutropenia and anemia. JAK2 is associated with homodimeric receptor complexes required for hematopoiesis. Phase II clinical trials with Abrocitinib have shown promising results with rapid improvement of clinical symptoms of AD and pruritus and very low rates of adverse effects [196,197].

Upadacitinib is a selective JAK1 inhibitor that was FDA approved in 2019 for the treatment of rheumatoid arthritis, which is an inflammatory disease. However, currently, phase III clinical trials are ongoing for potential use of Upadacitinib for treating moderate to severe AD. Phase II trials have shown promising results with good tolerability and low adverse effects with improvement of symptoms of AD from the first week of treatment. The study also indicated good clinical efficacy endpoints and significant reduction in pruritus [196,198].

Gusacitinib (*ASN002*): is the first dual JAK/SYK inhibitor to undergo clinical trials in AD patients. Spleen tyrosine kinase (SYK) is a non-receptor tyrosine kinase that inhibits terminal differentiation of keratinocytes and is involved in Th17/IL-17 signaling pathway and B cell and dendritic cell differentiation. JAK inhibitors target JAK1 and JAK2 signaling pathways, thereby modulating the cytokine axes involved in AD pathogenesis such as Th2, Th22, and Th1. Thus, the dual inhibition of JAK and SYK can widen the range of targeted cytokines and increase the clinical efficacy of JAK inhibition by resolving different subtypes of AD. Clinical trials with gusacitinib have indicated good tolerability and significant reduction in pruritus and inflammatory lesion count [199,200].

Adriforant (*ZPL-3893787*) is a high-potency small molecule capable of selectively inhibiting the histamine H4 receptor with almost 100-fold more specificity towards the H4 receptor than the H1, H2, and H3 receptors. H4 is the latest member of the histamine receptor family. It is widely expressed on a variety of immune cells including T cells, eosinophils, dendritic cells, and mast cells. Hence, it is also known as the “histamine receptor of the immune system” and is closely associated with several functional inflammatory responses regulated by histamine, such as modulation of cytokine and chemokine release, chemotaxis, cell recruitment, and upregulation of adhesion molecule expression. Histamine is also known to mediate itch and enhance pruritus in AD patients. Clinical trials with adriforant (ZPL-3893787) showed anti-inflammatory effects with good tolerability and low adverse events. However, wider studies should be pursued before it can be promoted for clinical use [201].

Serlopitant is a small molecule inhibitor of neurokinin 1 receptor (NK1R), which is expressed on various skin cell types such as fibroblasts, keratinocytes, and mast cells. NK1R is associated with inducing itch signals through a non-histaminergic pathway by interacting with its ligand, substance P (SP). Studies have shown that NK1R genes are upregulated in AD patients, and SP-expressing nerve fibers are increased in the pruritic skin. Clinical trials to evaluate serlopitant as a potential therapeutic for AD to reduce itching and pruritus indicated that serlopitant was capable to reduce the pruritus associated with AD. However, the results were not statistically significant, and further studies are required to establish clinical significance [202,203].

Tradipitant (*VLY-686*) is a small molecule inhibitor of the NK1 receptor that blocks this receptor and prevents interaction with its ligand, substance P (SP), which is released on activation of sensory neurons. NK1R–SP interactions result in transmitting itch signals to the central nervous system through a non-histaminergic pathway. Phase III clinical trials indicated a significant improvement in severe pruritus and sleep disturbance after one day of treatment [204].

Apremilast is a small molecule inhibitor of phosphodiesterase 4 (PDE4), an enzyme involved in cAMP hydrolysis. It is seen in keratinocytes, Langerhans cells, dendritic cells, monocytes, Th cells, and eosinophils. Inhibition of PDE4 results in failure to hydrolyze cAMP, resulting in the accumulation of cAMP in immune and non-immune cells and prevention of the release of pro-inflammatory mediators [205]. The use of oral PDE4 inhibitors such as Apremilast is in ongoing phase II clinical trials. It has shown up to 19 to 39% reduction of PDE4 in moderate to severe adult AD patients [206].

#### 8.3.3. Phototherapy

Extremely severe cases of AD with widespread body lesions that cannot be controlled by topical therapies such as TCSs may require UVB phototherapy to reduce symptoms such as pruritus. Even though this treatment does not have a risk of skin cancer [207], it may be inconvenient in terms of requiring 2–3 treatment sessions per week for a few months [7]. Phototherapy in combination with topical agents such as psoralen has shown to reduce pruritus within 2 weeks of treatment [208]. Phototherapy is basically UV radiation that helps in thickening the stratum corneum, which can then prevent the entry of foreign substances and inhibit *S. aureus* colonization through its anti-microbial properties. Phototherapy also helps to accomplish immunosuppression in the skin by targeting the resident immune cells by inducing apoptosis of infiltrating T cells, altering cytokine production and inhibiting the antigen-presenting function of Langerhans cells. Different types of light therapy of varying degrees have proven beneficial to AD patients, including narrow-band (NB)-UVB, broad-band (BB)-UVB, UVA, and more importantly UVA1 and UVB [209].

## 9. Conclusions

Despite being the most common among non-fatal skin diseases, the pathophysiology of AD is still poorly understood. Perspectives on this burdensome disease are continuously being evolved with advancement of our knowledge about the underlying molecular mechanisms. It is quite evident that AD is not just a skin condition but extends beyond to damaged sleep, disturbed social performances, and disruption to the overall development of the individual. The possibility of recognizing “at-risk” babies at birth may determine the success of AD management in the future. Hence, there is a need to understand more about different barrier components and how they are linked to immune regulation to optimize barrier enhancement approaches. Knowing that AD is a Th2-driven disease, recent therapeutic approaches aim to target pathways involved in pathophysiology such as by modulating Th2-related cytokines and receptors. Clinical and epidemiological researchers have aimed to more precisely define the different AD genotypes, novel pathways, and associated phenotypes that will prove an asset towards identifying novel biomarkers and personalized targets for better therapeutic approaches to improve the patient’s quality of life and health outcomes.

## Figures and Tables

**Table 1:** Emerging systemic therapeutic biologics (monoclonal antibodies) for AD.

Therapeutic Target	Drug Name	Mechanism of Action	AD Severity	Route of Administration	Status	Trial Identifier
IL-4Rα	Dupilumab	Inhibits the IL-4/IL-13 signaling pathway by blocking the shared IL-4 receptor-α subunit, thus reducing the Th2 response.	Moderate to severe	Subcutaneous injections	FDA approved in 2017	**Phase II** NCT03861455 NCT03346434 **Phase III** NCT02612454 NCT01949311 **Phase IV** NCT03667014 NCT03389893 NCT03293030 NCT04447417 NCT04033367
IL-13	Lebrikizumab	Inhibits IL-13 by preventing the formation of the IL-13Rα1/IL-4Rα heterodimer.	Moderate to severe	Subcutaneous injections	Completed	**Phase III** NCT04146363 NCT04178967 NCT04392154 NCT04250350 NCT04250337
IL-13	Tralokinumab	Binds with high affinity to IL-13, preventing its interaction with its receptors and succeeding signaling pathways.	Moderate to severe	Subcutaneous injections	Completed	**Phase III** NCT03587805 NCT03761537 NCT03526861
IL-31	Nemolizumab	Inhibits IL-31 by binding to its receptor α subunit (IL-31α).	Moderate to severe	Subcutaneous injections	Completed	**Phase II** NCT03921411 NCT04365387 **Phase III** NCT03985943 NCT03989349 NCT03989206
IL-22	Fezakinumab	Blocks IL-22 involved in skin hyperplasia and skin barrier dysfunction.	Moderate to severe	Intravenous injections	Completed	NCT01941537
IL-12/IL-23	Ustekinumab	Blocks IL-12 and IL-23 by targeting their common p40 subunit, thus inhibiting the Th1 and Th17/Th22 responses	Moderate to severe	Subcutaneous injections	Completed	NCT01806662
IL-33	Etokimab	Blocks IL-33 and hence effectively inhibits neutrophil migration.	Moderate to severe	Intravenous injections	Ongoing	NCT03533751
IgE	Omalizumab	Binds to the Fc (CH3 domain) of IgE molecules and prevents its interaction with its high affinity receptor FcεRI on mast cells and basophils, inhibiting the process of mast degranulation and release of inflammatory mediators.	Moderate to severe pediatric	Intravenous injections	Completed	NCT02300701
OX40	ISB 830	Binds to OX40 (CD135), a co-stimulatory molecule expressed on T cells, and prevents its interaction with OX40L (CD252) expressed on activated antigen presenting cells, thus failing to potentiate the T cell responses triggered through T cell receptors.	Moderate to severe	Intravenous injections	Completed	**Phase IIb** NCT03568162
TSLP	Tezepelumab	Binds to TSLP and prevents its interaction with its receptor hence blocking all downstream events associated with immune-modulating proteins and Th2 cytokines.	Moderate to severe	Subcutaneous injections	Completed	**Phase IIb** NCT02525094

**Table 2:** Emerging systemic therapeutic small molecules (pathway inhibitors) for AD.

Therapeutic Target	Drug Name	Mechanism of Action	AD Severity	Route of Administration	Current Status of Study	Trial Identifier
JAK1/2	Baricitinib	Inhibits JAK1 and JAK2 in the JAK-STAT signaling pathway, thus exerting an immunomodulatory and anti-proliferative effects. This is achieved by inhibiting immune cell function by detaching cytokine effects from the cells and	Moderate to severe	Oral	Ongoing phase III trials	**Phase II** NCT02576938 **phase III** NCT03334396 NCT03334422 NCT03428100 NCT03435081 NCT03733301 NCT03334435 NCT03559270
JAK1	Upadacitinib	Selectively inhibits JAK1 of the JAK-STAT pathway, thus blocking downstream cellular processes contributing to inflammatory conditions.	Moderate to severe	Oral	Ongoing phase III trials	**Phase II** NCT02925117 **Phase III** NCT03569293 NCT03568318 NCT03661138 NCT04195698 NCT03607422 NCT03738397
JAK1	Abrocitinib	Selectively inhibits JAK1 of the JAK-STAT signaling pathway, thus preventing activation of JAK1 containing heterodimeric receptors and thereby inhibiting downstream Th2 differentiation and itch while avoiding undesirable effects of JAK2 inhibition such as neutropenia and anemia.	Moderate to severe	Oral	Ongoing phase III trials	**Phase II** NCT02780167 **Phase III** NCT04345367 NCT03422822 NCT03627767 NCT03796676 NCT03720470 NCT03575871 NCT03349060
JAK/SYK	Gusacitinib (ASN002)	Targets a broad range of cytokine axes through dual inhibition of JAK and SYK, a non-receptor tyrosine kinase that inhibits terminal differentiation of keratinocytes and is involved in regulation of B cell and dendritic cell. differentiation and Th17/IL-17 signaling	Moderate to severe	Oral	Completed phase II trials	**Phase Ib** NCT03139981 **Phase II** NCT03728504 NCT03654755 NCT03531957
H4R	Adriforant (ZPL-3893787)	Highly potent;selectively inhibits histamine H4 receptor, thus blocking associated chemotactic and inflammatory responses in immune cells such as eosinophils, dendritic cells, and mast cells.	Moderate to severe	Oral	Completed	**Phase II** NCT02424253
NK1-R	Serlopitant	Binds to neurokinin 1 receptor (NK1R) expressed on keratinocytes, mast cells, and fibroblasts and prevents interaction of its ligand, substance P (SP). This blocks the itch signals transmitted through the non-histaminergic pathway.	Chronic pruritus	Oral	Terminated	**Phase II** NCT03841331 NCT03343639 NCT02975206 NCT02196324 NCT01951274 NCT03282591 NCT00835718 NCT00290563 **Phase III** NCT03540160 NCT03677401 NCT03546816
NK1-R	Tradipitant (VLY-686)	Inhibits the NK1 receptor and blocks SP- mediated itch signals.	AD with significant pruritus	Oral	Completed	**Phase II** NCT02004041 **Phase III** NCT03568331
PDE4	Apremilast	Inhibits phosphodiesterase4 (PDE4) hence preventing the hydrolysis of cAMP. This results in an increase in cAMP levels in immune and non-immune cells and helps to modulate the expression of various pro-inflammatory mediators.	Moderate to severe	Oral	Completed	**Phase II** NCT00931242 NCT02087943

## Data Availability

Not applicable.
